# Impact of sports nutrition intervention on seasonal changes in nutrition and body composition in NCAA athletes

**DOI:** 10.1080/15502783.2025.2550163

**Published:** 2025-08-30

**Authors:** Celia Lemieux, Ethan Werlau, Sophie Gulleyan, Mason Margut, Jamie McAllister-Deitrick, Kimberly Michelle Singleton

**Affiliations:** Coastal Carolina University, Department of Kinesiology, Conway, SC, USA

**Keywords:** Dietary behavior, sports, nutrition, body composition

## Abstract

**Background:**

It is well known that proper nutritional intake strategies are essential to meeting the physiological demands of athletic performance and to optimize positive training adaptations. Additionally, athletes have a higher nutritional demand due to their increased level of physical activity. Previous research has shown collegiate athletes present with insufficient levels of sport nutrition knowledge (SNK) and they typically overestimate their level of understanding. This poor SNK can lead to inadequate fueling strategies and failure to meet sport-specific guidelines on nutritional intake. Therefore, the aim of the present study was to evaluate the effects of a sport nutrition education intervention (SNEI) on body composition among NCAA Division I (DI) athletes throughout a sports season.

**Methods:**

Thirty-two NCAA Division I soccer players (female, *n* = 19; male, *n* = 13) participated in the study during in-season training/matches. The intervention consisted of a detailed sports nutrition guide highlighting macronutrients, micronutrients, hydration, and supplementation, as well as recommendations for energy and macronutrient intake. Athletes received the SNEI at baseline.

**Results:**

Paired samples t-test revealed no significant differences in body mass (*p* = 0.526), body fat percentage (*p* = 0.817), fat-free mass (*p* = 0.465), and fat mass (*p* = 0.165).

**Conclusions:**

In this pre- post-intervention study evaluating body composition, the maintenance of overall body mass, body fat percentage, fat-free mass, and fat mass among DI female and male athletes indicate the SNEI positively impacted dietary behavior. Further research on SNEI is recommended, specifically assessing actual dietary intake and exploring interventions through a theoretical approach to gain a better understanding of an athlete’s dietary behavior.

